# Climate, Humidity, and Population-Level Interest in Dry Skin: Infodemiology Analysis Using Google Trends Across the United States

**DOI:** 10.2196/93639

**Published:** 2026-05-25

**Authors:** Kimiya Aframian, Shaya Naimi, Joy Xu, Gordon Bae

**Affiliations:** 1 Department of Dermatology, Stanford Medicine, Stanford, CA, United States; 2 Department of Sciences, CU Boulder Aerospace Engineering Sciences, Boulder, CO, United States; 3 Department of Medicine, David Geffen School of Medicine, 10833 Le Conte Ave, Los Angeles, CA, 90024, United States, 1 3109481476

**Keywords:** xerosis, dry skin, humidity, dew point, temperature, google trends, infodemiology, United States

## Abstract

**Background:**

Climate and weather factors of temperature and humidity are widely reported to be associated with xerosis (dry skin), a common inflammatory skin condition and frequent driver of pruritus (itchy skin) and reduced quality of life. Growing evidence supports links between environmental conditions and skin barrier function, with extreme climates associated with increased atopic dermatitis–related clinical visits. Mechanistically, temperature and humidity affect the stratum corneum, the skin’s primary permeability barrier, with low humidity and high temperature increasing transepidermal water loss and promoting cutaneous inflammation.

**Objective:**

This study examines the relationship between climate, namely temperature and humidity, and the general public’s experience in dry skin and moisturizing products, throughout the United States. This study sought to address gaps in traditional epidemiologic approaches by linking climate conditions with population-level online search behavior related to dry skin and moisturizer use across the United States.

**Methods:**

Publicly available climate data were obtained from the National Oceanic and Atmospheric Administration (NOAA), including average temperature and dew point by state over a recent nine-year period (2016‐2025). Dew point served as a proxy for ambient humidity. Google Trends was used to assess relative search interest for five dry skin– and moisturizer-related terms by state during the same period. Search interest was normalized per million residents, and associations between climate variables and search interest were evaluated using linear regression analyses. Statistical analyses were conducted using R.

**Results:**

Lower average temperatures and lower dew points were associated with higher dry skin–related search interest, while warmer, more humid states showed lower interest. Both temperature and dew point demonstrated significant negative associations with Google search interest. This work was not funded and data collection was performed using publicly available, free databases.

**Conclusions:**

Population-level search behavior related to xerosis reflects national patterns of climate-associated dermatologic burden.

## Introduction

Climate and weather factors of temperature and humidity are widely reported to be associated with xerosis (dry skin), one of the most common inflammatory skin conditions and frequent drivers of pruritus and reduced quality of life [1]. Although the mechanism is not fully understood, there is a widely growing body of evidence supporting meaningful links between environmental conditions and skin barrier function; regions characterized by extreme environmental conditions (temperature variability, precipitation patterns, sunlight exposure, and airborne pollutants) are associated with higher rates of atopic dermatitis (AD)–related clinical visits [2,3].

Mechanistically, temperature and humidity directly affect the stratum corneum, the skin’s primary permeability barrier of highly organized extracellular lipids which limit transepidermal water loss (TEWL) [4]. Factors that increase evaporation from the stratum corneum, such as low humidity and high temperature, impair corneocyte cohesion and promote cutaneous inflammation. Prior studies demonstrate the impacts of dry environment on increasing TEWL and impaired barrier repair, and high humidity on barrier restoration [5]. Such effects may be amplified in individuals predisposed to xerosis and eczematous dermatoses who already have baseline defects in barrier function [6]. Thus, “cold and dry” conditions may increase AD prevalence and flare risk, and cohort data suggests that climate factors may modulate symptom control in potentially region-specific methods [7].

Moisturizer is the mainstay management for xerosis and AD, so climate variability may plausibly be reflected in variation in skin-care needs on a regional-scale. Traditional epidemiologic analysis of the environment-skin relationship often relies on clinic visits, prescription, and registry records, however these methods do not represent subclinical symptoms and self-management behaviors outside of the formal healthcare environment [8]. Especially with the increase in accessibility to freely-available medical information −83.4% of patients in 2020 report using the Internet prior to their dermatologic appointment - it is critical to consider new metrics of analysis [9].

Google Trends is a freely accessible analytics platform which allows for assessment of Google search query volumes and has increasingly been used as an infodemiologic tool to character population-level health behaviors [10]. Search activity is reflective of self-triage and self-treatment efforts, both before and after clinical visits, offering a potential window into symptom burden captured beyond administrative data [11]. Specifically in dermatologic epidemiology, Google Trends data has been analyzed on a global scale to study temporal and geographic patterns of incidence, symptoms, and treatment of common conditions including eczema, acne, psoriasis, and skin cancer [12].

The aim of this paper is to understand the relationship between climate conditions and population-level interest in dry skin symptoms through linking of NOAA-reported temperature and humidity (dewpoint used as proxy) with Google Trends search interest across each US state for moisturizer-related and dry-skin symptom terms.

## Methods

### Google Trends Data

**Figure 1.**
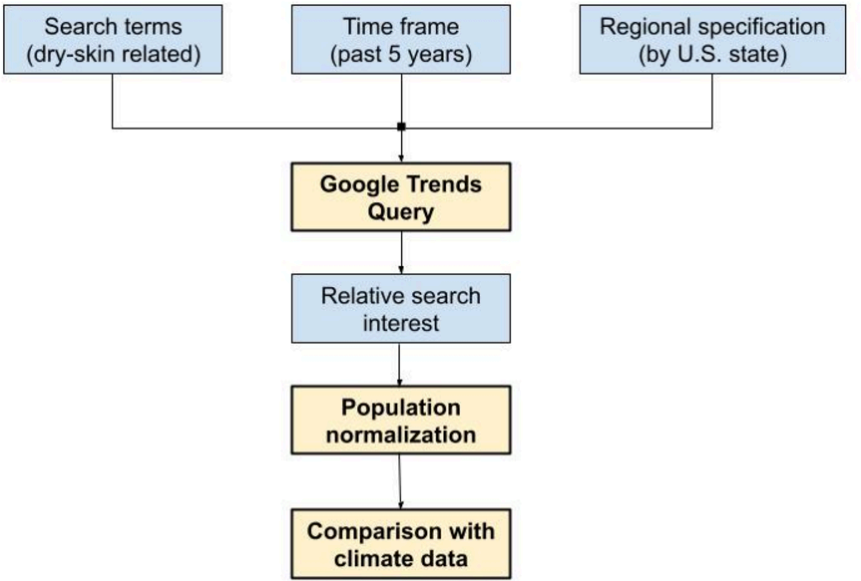
Outline of methodology. Google Trends query parameters were gathered, and the output of the query was normalized by state population prior to comparison against climate data.

The Google Trends software allows the public to query the search interest of one or more particular search terms over a user-specified period of time and region, and allows the user to compare regions, time frames, and search terms (Figure 1). When queried, the software tool yields relative search interest between regions (eg, US states), normalized between 0 and 100 across the data set, as opposed to absolute search volume. According to this scale, a value of 0 indicates the lowest search popularity and a value of 100 indicates the highest relative search interest amongst the data set. To obtain data for this study, the Google Trends software tool was utilized to query search interest in five search terms related to dry skin and moisturizer: “eczema,” “dry skin,” “moisturizer,” “atopic dermatitis,” and “atopic eczema” over the same 9-year period specified above for climate data and by state. Relative search interest was normalized by each state’s population. Note that this additional step of population normalization is independent of the “normalized” data provided by each Google Trends query; Google Trends provides relative search interest normalized against the highest value in the dataset, which does not involve population. To normalize by state population, each state’s relative interest was divided by its population to obtain “relative search interest per million people” (Figure 1).

### Climate Data

Publicly available data were obtained from the National Oceanic and Atmospheric Administration (NOAA), a US government agency that studies the earth’s oceans, atmosphere, and climate and publishes robust datasets available to the public. In particular, the NOAA offers the Climate Data Online (CDO), a web page that provides access to global historical weather and climate data, including daily, monthly, and hourly measurements of temperature, humidity, wind, and precipitation [13]. For this study, hourly measurements of average temperature and dew point by state were extracted over a recent 9-year period (2016‐2025). Note that dew point is a widely known excellent and often preferred proxy for humidity, as it measures air moisture more directly and independently of temperature [14]. For each state, these measurements were averaged over the recent 9-year period to yield average temperature and dew point by state over the given time frame.

## Results

### Google Trends Data

**Figure 2.**
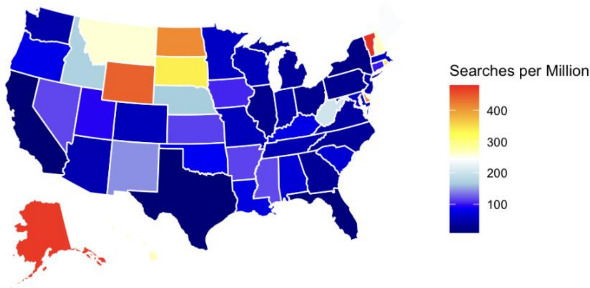
Google Trends Search Interest per capita by US state 2016‐2025 period. This map was generated using data from the Google Trends software tool.

Analysis of Google Trends data at the state level demonstrates substantial geographic variability in search interest for moisture-related and dry skin-associated search terms across the U.S. Higher relative search interest was observed in western and southwestern states, whereas lower search interest was observed in northeastern and midwestern states (Figure 2). Note that Google search interest was normalized per million residents, accounting for variability in population per state.

### Climate Data

**Figure 3.**
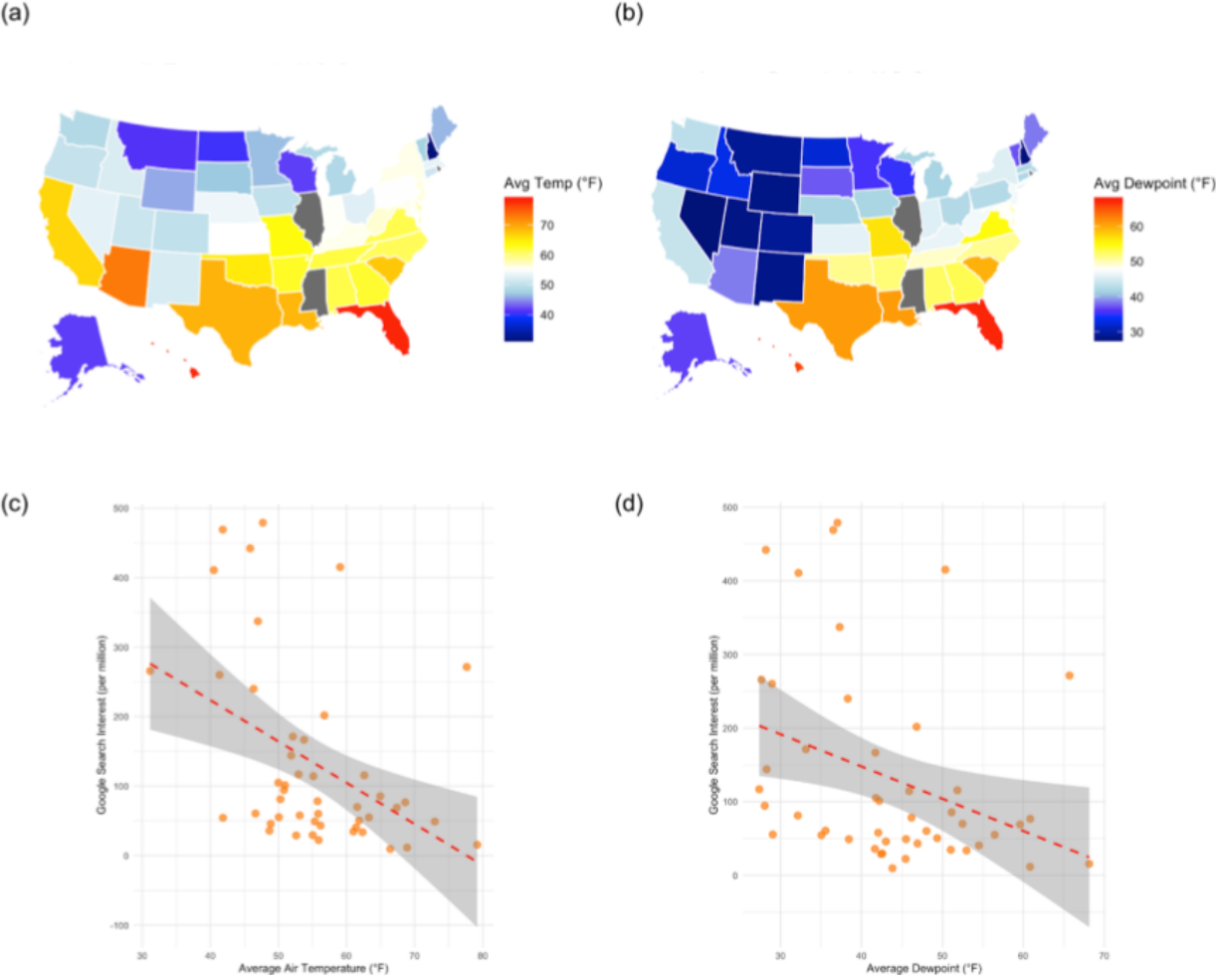
(A) Average air temperature by US state over 2016‐2025 period. (B) Average dewpoint by US state over 2016‐2025 period. (C) Plot illustrating the relationship between average dry bulb temperature (air temperature) and Google search popularity, averaged over the 2016‐2025 period, where each point represents one US state. There is a significant downward trend with Pearson *r*=-0.442 and *P*=.002. (D) Plot illustrating the relationship between average dewpoint (proxy for humidity) and Google search popularity, averaged over the 2016‐2025 period, where each point represents one US state. There is a significant downward trend with Pearson *r*=-0.348, and *P*=.02. All plots were generated using data from the National Oceanic and Atmospheric Administration.

Comparison of the Google Trends data with NOAA-reported climate variables reveal that states such as Vermont and Wyoming, among those with the highest search interest, generally exhibited cooler average temperatures and moderate dew point values, although variation across states was observed. (Figure 3A and C). Examining temperature at the state level, Google search interest (mean 132.79, SD 132.99) was negatively correlated with Average Air Temperature (°F) (mean 55.35, SD 9.8), r(45) = −0.428, 95% CI [−0.637,−0.16], *P*=.002. Statistical analyses were conducted using R, and regression assumptions, including linearity and normality of residuals, were assessed using visual inspection of diagnostic plots.

Average dewpoint is used as a proxy for ambient humidity, showing a similar pattern as temperature on a national level. States with a lower average dewpoint and thus presumably decreased humidity conditions, especially western interior states, exhibited higher relative search interest for moisturizers and dry skin-related symptoms (Figure 3B). Contrarily, states with higher average dewpoints such as the humid southeastern states demonstrated lowest search interest in these search terms. Average dewpoints ranged from 28 degrees Fahrenheit minimum to 68 degrees Fahrenheit maximum. Similarly to temperature, at the state level, Google search interest (M=132.79, SD=132.99) was negatively correlated with Average Dewpoint (°F) (mean 43.5, SD 10.51), r(45) = −0.338, 95% CI [−0.57,−0.056], *P*=.02. (Figure 3D).

## Discussion

### Principal Findings

This study found a significant negative correlation between average air temperature and Google search interest in dry skin-related terms per million residents, as well as a significant negative correlation between average dewpoint and Google search interest.

Regional humidity, rather than temperature alone, more closely aligns with patterns of dermatologic search interest according to geographic visualization of climate variables. Western inland states are characterized by lower average dewpoints and higher evaporation demand. These states consistently exhibit elevated Google Trends search interest in our queried terms. In contrast, coastal and southeastern states with high relative humidity and high dewpoints showed lower search interest despite the higher acreage annual temperatures. These findings suggest that atmospheric moisture content, represented by dew point, may be an important contributor to patterns of dermatologic search interest. While these findings demonstrate an association rather than causation, possible mechanisms underlying this relationship have been proposed. For example, temperature and humidity have been shown to influence skin barrier integrity, whereby low humidity may increase evaporation from the skin surface and contribute to skin dryness.

These findings cumulatively represent a robust association between climate conditions and Google search behavior related to moisturizer and dry skin symptoms across the United States. To our knowledge, this study represents an early effort to use Google Trends data as a digital epidemiologic tool to explore geographic variation in dry-skin related search activity across the United States. While search intent does not directly equate to disease prevalence, this approach provides a means of visualizing regional patterns, including clusters of elevated interest that are frequently observed in coastal areas. Google trends has also successfully predicted the outbreak of many viruses [15], indicating its validity as an epidemiological proxy.

### Limitations

Limitations of this study include the difference in population per state. Normalization by population in search interest is reasonably assumed to be proportional to population, but states with extremely high or low populations may have resulted in skewed relative search interest per capita. Populations with limited internet access and healthcare utilization as well as lower socioeconomic status may be poorly represented. Demographic differences between states may also account for differences in data. Additionally, Google trends queries return relative search interest normalized between 0‐100 across the data set instead of absolute search volumes, regardless of the data set being queried. Thus, reported findings are correlational and cannot be assumed as causation. In addition, analysis used multiyear averaged data, which may obscure important seasonal variation in skin dryness and related search behavior. These temporal patterns may not be fully captured in the present approach.

### Conclusion

This study finds a strong correlation between NOAA reported temperature and humidity and state-specific Google searches, revealing that online search behavior reflects the general public’s experience of the climate across the states. In other words, Americans who live in hotter and drier states are more likely to seek out moisturizer and to experience symptoms of dry skin conditions such as eczema and dermatitis. These findings reveal the potential for Google trends to monitor the general public’s awareness of their dermatological response to varying climates, and can ultimately allow physicians to tailor preventative skin-care recommendations and emphasize proactive skin barrier protection in more predisposed environments. These findings highlight the potential for infodemiology tools to complement traditional dermatologic surveillance and may help guide future studies on environmental influences on skin disease.

Furthermore, physicians and medical systems at large could consider prioritization of access to moisturizers and barrier-rapid therapy in higher-risk regions with anticipated increased xerosis burden.

## Abbreviations

AD: atopic dermatitis
NOAA: National Oceanic and Atmospheric Administration
TEWL: transepidermal water loss
